# Tissue recommendations for precision cancer therapy using next generation sequencing: a comprehensive single cancer center’s experiences

**DOI:** 10.18632/oncotarget.17199

**Published:** 2017-04-18

**Authors:** Minho Cho, Soomin Ahn, Mineui Hong, Heejin Bang, Michael Van Vrancken, Seungtae Kim, Jeeyun Lee, Se Hoon Park, Joon Oh Park, Young Suk Park, Ho Yeong Lim, Won Ki Kang, Jong-Mu Sun, Se Hoon Lee, Myung-Ju Ahn, Keunchil Park, Duk Hwan Kim, Seunggwan Lee, Woongyang Park, Kyoung-Mee Kim

**Affiliations:** ^1^ Center for Cancer Companion Diagnostics, The Innovative Cancer Medicine Institute, Samsung Medical Center, Seoul, Korea; ^2^ Department of Pathology and Translational Genomics, Samsung Medical Center, Sungkyunkwan University School of Medicine, Seoul, Korea; ^3^ Department of Pathology and Laboratory Medicine, Tulane University School of Medicine, New Orleans, Louisiana, USA; ^4^ Department of Medicine, Division of Hematology-Oncology, Samsung Medical Center, Sungkyunkwan University School of Medicine, Seoul, Korea; ^5^ Medical Translational Research Center, Samsung Biological Research Institute, Seoul, Korea; ^6^ Department of Integrated Health and Environmental Science, College of Health Science, Korea University, Seoul, Korea; ^7^ Samsung Genome Institute, Seoul, Korea; ^8^ Present address: Department of Integrated Health and Environmental Science, College of Health Science, Korea University, Seoul, Korea

**Keywords:** cancer, DNA, next generation sequencing, therapy, target

## Abstract

To generate accurate next-generation sequencing (NGS) data, the amount and quality of DNA extracted is critical. We analyzed 1564 tissue samples from patients with metastatic or recurrent solid tumor submitted for NGS according to their sample size, acquisition method, organ, and fixation to propose appropriate tissue requirements.

Of the 1564 tissue samples, 481 (30.8%) consisted of fresh-frozen (FF) tissue, and 1,083 (69.2%) consisted of formalin-fixed paraffin-embedded (FFPE) tissue. We obtained successful NGS results in 95.9% of cases. Out of 481 FF biopsies, 262 tissue samples were from lung, and the mean fragment size was 2.4 mm. Compared to lung, GI tract tumor fragments showed a significantly lower DNA extraction failure rate (2.1 % versus 6.1%, *p* = 0.04). For FFPE biopsy samples, the size of biopsy tissue was similar regardless of tumor type with a mean of 0.8 × 0.3 cm, and the mean DNA yield per one unstained slide was 114 ng. We obtained highest amount of DNA from the colorectum (2353 ng) and the lowest amount from the hepatobiliary tract (760.3 ng) likely due to a relatively smaller biopsy size, extensive hemorrhage and necrosis, and lower tumor volume. On one unstained slide from FFPE operation specimens, the mean size of the specimen was 2.0 × 1.0 cm, and the mean DNA yield per one unstained slide was 1800 ng.

In conclusions, we present our experiences on tissue requirements for appropriate NGS workflow: > 1 mm^2^ for FF biopsy, > 5 unstained slides for FFPE biopsy, and > 1 unstained slide for FFPE operation specimens for successful test results in 95.9% of cases.

## INTRODUCTION

The molecular assessment of malignant tumors has become increasingly important in recent years in this era of precision cancer therapy and personalized medicine. Such studies typically give information regarding possible therapeutic targets as well as important prognostic information. Recent advances in high-throughput next-generation sequencing (NGS) technology have substantially reduced the cost and increased the workflow speed in helping to detect important genetic alterations [1, 2].

There are multiple quality control checkpoints throughout the NGS process [3]. Regardless of the NGS platform or exact method used, one of the first steps involved in NGS is tissue preparation and DNA extraction. The importance of the quality of the DNA extracted cannot be overemphasized as it affects the subsequent sequencing quality and final results. Although fresh frozen (FF) tissue is preferred over formalin-fixed paraffin-embedded (FFPE) tissue, FFPE is typically used more due to practical concerns. The amount and quality of starting DNA used for NGS depends mainly on the desired application or workflow to create the appropriate library. The Illumina platform usually requires > 100 ng of DNA input, and the initial fragment size range is recommended to be between 150–200 base pairs in length.

For tumor volume, it is widely accepted that specimens with < 10% tumor are not eligible for NGS because sequencing of samples with lower tumor percentages may cause difficulty in detecting copy number variation (CNV) and distinguishing true variants from sequencing artifacts [4]. Moreover, intratumoral heterogeneity is typically underrepresented in these samples [5].

In clinical practice, tissue is routinely obtained through a biopsy procedure, however, like any procedure there are risks associated with it. In certain patients, the risk of complications can be quite high, particularly with those in which hemorrhage is a likely - such as tumors in the lung. Getting enough tissue for all the studies needed for these patients can be quite challenging. Information regarding DNA yield in these small biopsies is limited due to the newness of the technology and the fact that it has not been used much in the clinical laboratory setting [6–10]. During the last three years at our institution, we have performed targeted NGS for palliative targeted therapy on a large number of tissue samples giving us valuable experience in how to best utilize the limited resources often involved. Out of 1,564 patients that were analyzed, we acquired meaningful genomic results in 1,503 of them. From this experience, here we suggest appropriate tissue requirements to optimize NGS workflow for better clinical service and patient care.

## RESULTS

### DNA extraction from fresh frozen biopsy tissue

A total of 481 FF biopsy samples were submitted for sequencing. The mean tissue size and DNA amount according to recurrent or metastatic tumor and procedure type are summarized in Table 1. Overall, small tissue fragments with tissue volumes less than 8mm^3^ comprised 29.1% (140 of 481) of FF tissue. After pathologic examination, 21 out of the 481 cases were excluded due to no tumor (*N* = 3) or low tumor cell content (< 5%; *N* = 18).

**Table 1:** The average tissue size and DNA amount according to recurrent or metastatic tumor and procedure type

Biopsy sites	Biopsy methods	No. of cases	Average fragment size (mm) (range)	Average fragment numbers (range)	Average volume (mm^3^) (range)	Number of tissue volume < 8 mm^3^ (%)	Average tumor purity (%) (range)	Number of successful DNA extraction (%)	Average DNA yield/8mm^3^	Average total DNA amount (ng) (range)
GI tracts(*N* = 193)	Gastroscopic biopsy	104	2.3 (1.5 ~ 4.5)	2.9 (1 ~ 7)	33.3 (4 ~ 210)	9 (9)	61.0(5 ~ 95)	102 (98.1)	893.5	3720.8 (200 ~ 14,470)
	Colonoscopic biopsy	48	2.6(1.7 ~ 5.5)	2.3 (1 ~ 7)	33.2 (8 ~ 224)	5 (11)	57.4(10 ~ 90)	46 (95.8)	1023	4251.4(340 ~ 22,600)
Liver(*N* = 41)	Needle biopsy	41	2.1(1.0 ~ 5.0)	1.7 (1 ~ 4)	17.0 (2 ~ 144)	24 (59)	46.7(10 ~ 90)	41 (100)	603.5	1280.8(160 ~ 2,850)
Lung(*N* = 262)	Transthoracic needle aspiration biopsy	81	2.3(0.5 ~ 4.9)	1.7 (1 ~ 10)	20.3 (1 ~ 216)	38 (49)	56.1(5 ~ 95)	77 (95.1)	512	1299.9(120 ~ 8,230)
	Ultrasound guided biopsy	72	2.2 (1.2 ~ 5.0)	1.5 (1 ~ 6)	15.4 (3 ~ 162)	30 (43)	56.9(5 ~ 95)	70 (97.2)	807.2	1552.5(140 ~ 6,360)
	Bronchoscopic biopsy	51	2.3(1.2 ~ 3.9)	2.7 (1 ~ 5)	34.9 ( 1 ~ 10)	10 (22)	59.2(5 ~ 95)	46 (90.2)	657.3	2870.4(260 ~ 12,170)
	Endobronchial ultrasound bronchoscopy	40	3.8 (1.5 ~ 4.7)	2.9 (1 ~ 6)	76.5 (4 ~ 192)	5 (14)	51.3(5 ~ 90)	35 (87.5)	206	1971.8 (100 ~ 10,340)
	Gun biopsy	18	2.4 (1.2 ~ 4.3)	2.6 (1 ~ 8)	34.1 (3 ~ 216)	9 (50)	57.8(10 ~ 90)	18 (100)	407.8	1736.1 (280 ~ 6,220)
Skin	Punch biopsy	6	3.3 (2.5 ~ 4.5)	2.6 (1 ~ 4)	58.7 (10 ~ 160)	0 (0)	63.3(30 ~ 90)	6 (100)	563.7	4134.5 (330 ~ 6,830)
Others	Biopsy from lymph node, renal, soft tissue, bone	20	2.1 (0.7 ~ 4.5)	1.3 (1 ~ 4)	12.8 (1 ~ 96)	10 (53)	57.8(5 ~ 99)	19 (95)	818.8	1309.1 (110 ~ 5,100)
Total		481	2.4 (0.7 ~ 5.5)	2.4 (1 ~ 10)	29.5 (1 ~224)	140 (29)	56.8(5 ~ 99)	460 (95.6)	651.7	2431.2 (100 ~ 22,600)

Out of 481 FF biopsies, 262 tissue samples were from the lung. Tissue acquisition methods for lung biopsy consisted of transthoracic needle aspiration biopsy (*N* = 81), ultrasound guided biopsy (*N* = 72), bronchoscopy (*N* = 51), endobronchial ultrasound bronchoscopy (EBUS) (*N* = 40), and gun biopsy (*N* = 18). The mean size of all 262 lung tissue fragment was 2.4 mm (range 0.5 to 5.0) and tumor volume was 56.35% (range 5 to 95%, median 60%). In 246 lung cases (93.9%), enough DNA was extracted to successfully run NGS. The size of the smallest sample successfully run was 1 mm^2^.

193 FF tissue samples were from GI tract and consisted of endoscopic biopsies from the stomach (*N* = 104) and colorectum (*N* = 48) as well as computed tomography-guided liver biopsy specimens (*N* = 41). The mean size of the GI tissue fragments was 2.4 mm, and tumor volume was 60.68 (range 5 to 95). In 189 GI cases (97.9%), enough DNA was extracted to successfully run NGS. The size of the smallest sample successfully run was 4mm^2^. Compared to lung, GI tract tumor fragments showed a significantly lower DNA extraction failure rate (2.1 % *versus* 6.1%, *p* = 0.04).

**Figure 1:**
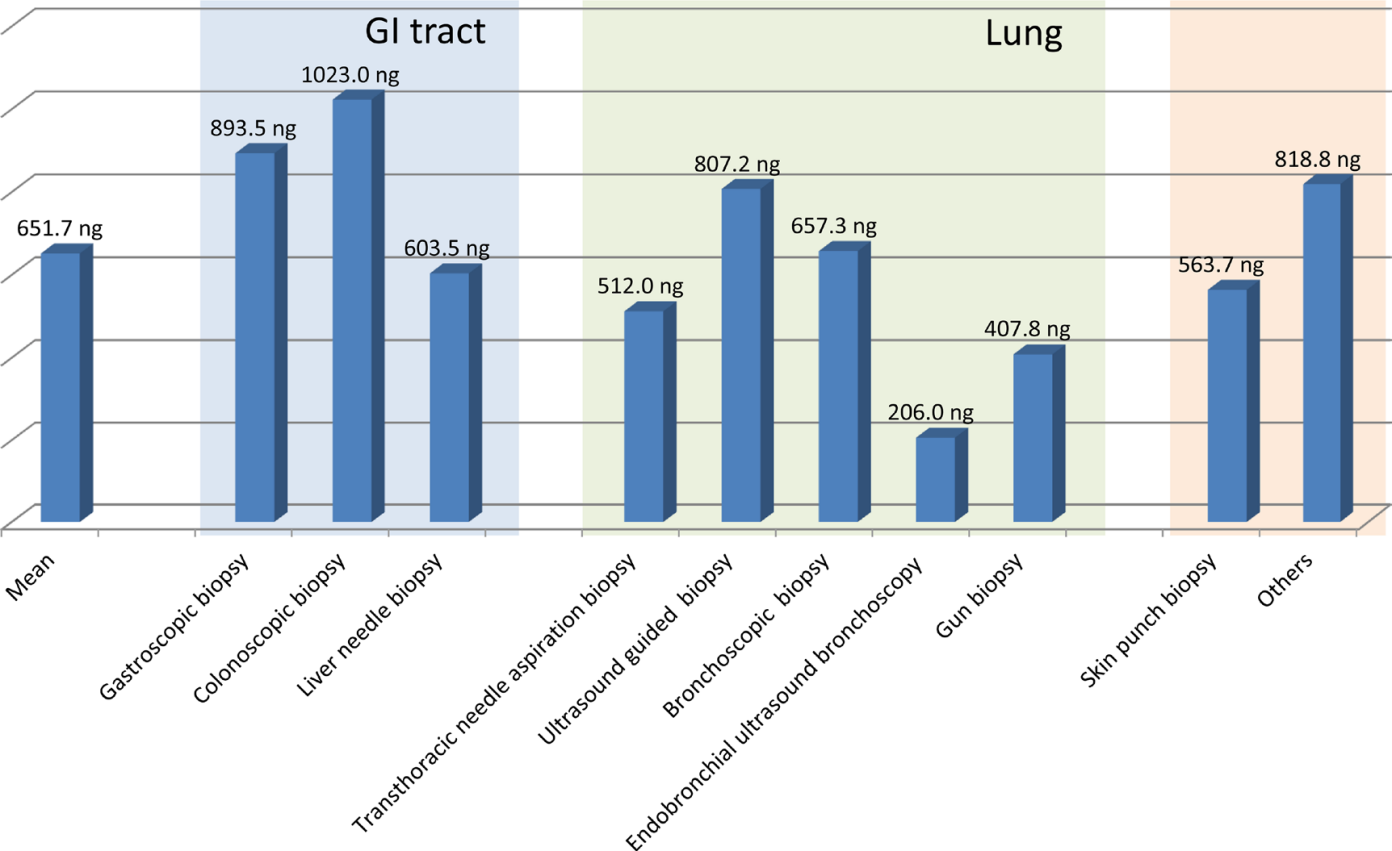
The mean DNA amount extracted from fresh frozen biopsy sample from different organs after adjustment to an endoscopic biopsy size (8 mm^3^)

The mean DNA volume was 2431.2 ng as measured by Nanodrop. DNA amounts according to cancer sites and acquisition method were also compared. For size, yield of DNA per one fragment of endoscopic biopsy was calculated in a volume of 8 mm^3^. The mean DNA yield from one endoscopic biopsy piece (ng/8 mm^3^) was 651.7 ng. The mean DNA yield was generally low in the specimens from lung compared to those of the GI tract (Figure 1). Interestingly, the highest DNA yield was acquired from colonoscopic biopsies (1023 ng/8 mm^3^) followed by gastric biopsy (893.5 ng/8 mm^3^) and ultrasound guided lung biopsy (807.2 ng/8 mm^3^). The lowest DNA amount was obtained from EBUS lung biopsy (206 ng/8 mm^3^) (Figure 1). As expected, skin biopsy showed no failure in acquisition of DNA.

### DNA extraction from FFPE tissue

Of 1083 FFPE samples, 341 were from biopsy material and 702 were from surgical specimens. 40 (3.7%) cases showed no tumor or low tumor percentage (< 10%) and were excluded from further DNA extraction.

**Figure 2:**
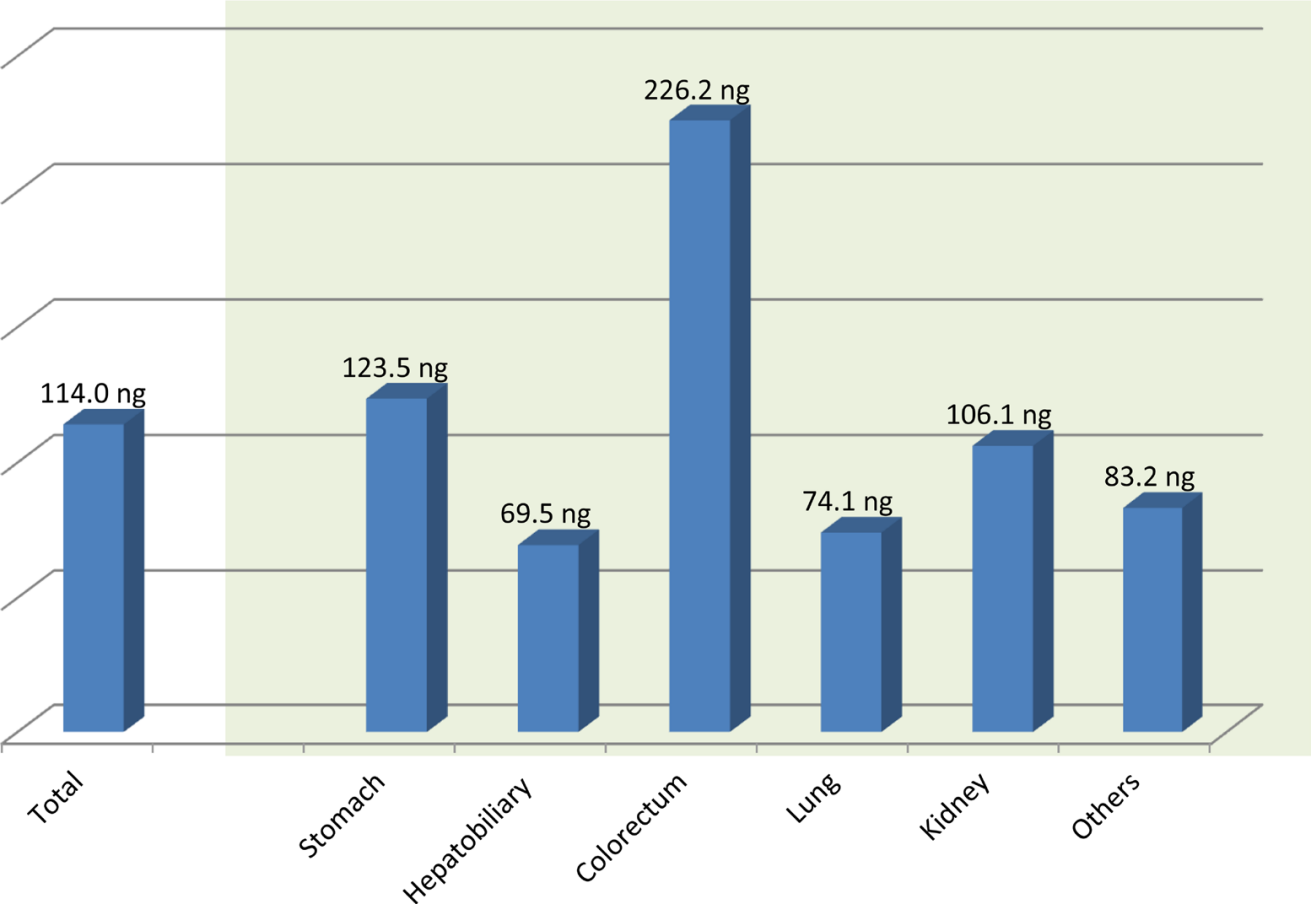
The mean DNA amount extracted from formalin-fixed, paraffin-embedded biopsy specimens per one 5 μm unstained section

Biopsy sites included the upper GI tract (*N* = 177), hepatobiliary tract (*N* = 59), colorectum (*N* = 33), lung (*N* = 36, kidney (*N* = 11) and others (*N* = 25). The size of biopsy tissue was similar in all tissue regardless of acquisition organ with a mean size of 0.8 × 0.3 cm. The mean DNA yield per one unstained slide was 114ng. To get 600 ng of DNA as measured by Nanodrop, we used on mean 11.5 unstained slides for DNA extraction in the biopsy samples. The mean DNA amounts from all FFPE biopsy samples were 1313 ng. According to acquisition organ, we procured the highest amount of DNA from the colorectum (2353 ng on mean) and the lowest amount from the hepatobiliary tract (760.3 ng on mean) (Figure 2). This is due to the hepatobiliary tract biopsies being on mean slightly smaller with lower tumor volume and typically having more extensive hemorrhage and necrosis. The tissue size and DNA amount extracted from FFPE biopsy samples according to acquisition site and tumor volume are demonstrated in Table 2. In FFPE biopsy samples, the smallest biopsy in which DNA was successfully extracted was 2 × 1mm (2 mm^2^), and 10 unstained slides were used for this case.

**Table 2:** Tissue size and DNA amount of FFPE biopsy samples according to acquisition site, tumor volume and numbers of slides

Biopsy sites	Number of cases	Mean sum size of biopsy (mm) (range)	Smallest biopsy		Tumor Volume (%) (range)	Average DNA yield/1 unstained slide (ng) (range)	Average Numbers of used slides (N) (range)	Average total DNA yields (ng) (range)
			sum size of biopsy (mm^2^)	Numbers of used slides				
Stomach	177	7 × 3 (2 × 1 ~ 10 × 10)	38	19	54 (5 ~ 90)	123.5 (2.7 ~ 396.5)	11.7 (4 ~ 19)	1445 (25.0 ~ 3706.0)
Hepatobiliary	59	8 × 3 (2 × 1 ~14 × 10)	18	9	43 (5 ~ 100)	69.6 (1.3 ~ 529.5)	10.93 (2 ~ 19)	760.3 (12.0 ~ 3177.0)
Lung	36	8 × 3 (2 × 1 ~ 18 × 10)	16	8	51 (10 ~ 95)	74.2 (3.1 ~ 767.6)	13.11 (3 ~ 19)	972.1 (59.4 ~ 3070.0)
Colorectum	33	10 × 3 (2 × 2 ~ 10 × 10)	36	9	55 (7 ~ 90)	226.2 (54.4 ~ 451.1)	10.4 (6 ~ 19)	2353.4 (489.6 ~ 4060.0)
Kidney	11	13 × 3 (2 × 1 ~ 15 × 10)	18	9	48 (6 ~ 80)	106.2 (3.3 ~ 382.8)	10.2 (4 ~ 19)	1090.2 (29.9 ~ 2290.0)
Others	25	5 × 3 (2 × 1 ~ 12 × 11)	38	19	54 (6 ~100)	83.3 (1.8 ~ 372.2)	10.8 (4 ~ 19)	902.8 (16.0 ~ 3696.0)
Total	341	8 × 3 (2 × 1 ~ 18 × 10)	16	8	52 (5 ~ 100)	114.0 (1.3 ~ 767.6)	11.5 (2 ~19)	1313.3 (12.0 ~ 4060.0)

**Figure 3:**
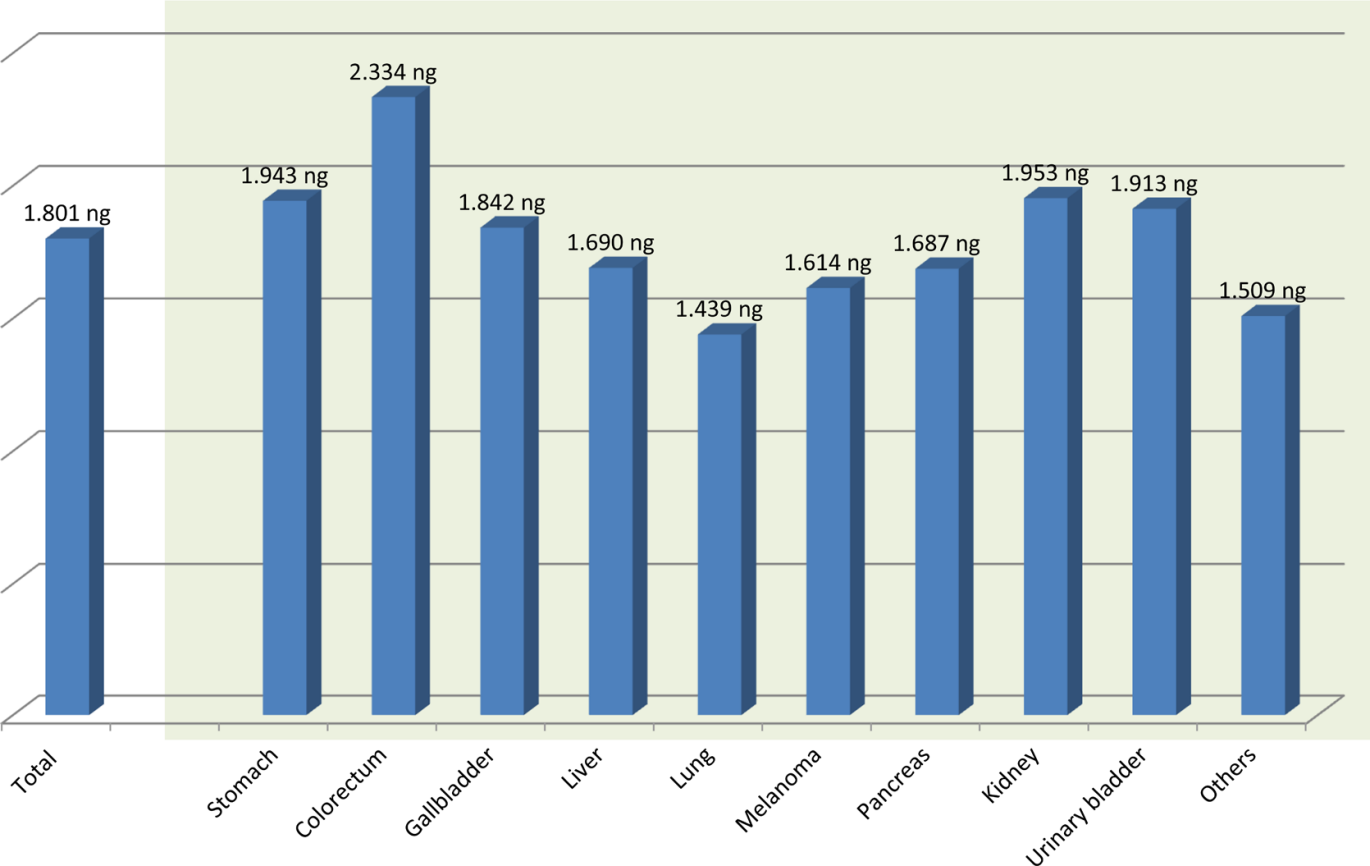
The mean DNA amount extracted from formalin-fixed, paraffin-embedded surgical samples per one 5 μm unstained section

For the larger specimens obtained after surgery, the mean size of the specimen received was 2.0 × 1.0 cm, and the mean DNA yield per one unstained slide was 1800 ng. Therefore, theoretically only one unstained section of operation specimen would be enough for NGS if properly fixed. However, due to our lack of experience, 7.2 unstained slides were used far exceeding the minimum amount of tissue needed for successful DNA extraction. According to acquisition organ, the highest amount of DNA was obtained from the colorectum (2334 ng on mean), and the lowest amount was obtained from lung specimens (1439 ng on mean) (Table 3 and Figure 3). The difference is likely attributable to extensive hemorrhage and necrosis in the lung specimens as well as varying operation room protocols in how specimens are treated and triaged. For colorectal, gastric, and renal cell carcinomas, the specimens are immediately sent to the pathology department after removal for prompt fixation. For lung cancer cases, the relatively long operational time likely increases the cold ischemia time in the tumor after removal. This undoubtedly affects the amount and quality of the DNA that can be successfully extracted.

**Table 3:** Tissue size and DNA amount of FFPE operation samples according to acquisition site and tumor volume

Primary tumor locations	Numbers	Average size of tumor in 1 slide (cm) (range)	Average area of tumor in 1 slide (cm^2^) (range)	Average tumor Volume (%) (range)	Average DNA amount from 1 unstained slide (ng) (range)
Stomach	141	2.3 × 1.0(1.0 × 0.3 ~ 3.5 × 2.5)	2.32(0.30 ~ 8.75)	54 ( 10 ~ 95)	1.943(14.6 ~ 8607.3)
Colorectum	101	2.0 × 1.0(0.7 × 0.5 ~ 2.5 × 2.5)	2.08(0.36 ~ 6.25)	55 (6 ~ 100)	2.334(260.0 ~ 9044.0)
Gallbladder	19	2.0 × 1.01.0 × 0.6 ~ 3.5 × 1.2)	2.00(0.60 ~ 4.20)	55 (10 ~ 90)	1.842(330.0 ~ 4181.0)
Liver	76	2.3 × 1.0(1.9 × 0.1 ~ 3.5 × 2.5)	2.36(1.90 ~ 8.75)	56 (10 ~ 95)	1.690(156.0 ~ 7816.0)
Lung	90	1.7 × 1.0(0.6 × 0.3 ~ 3.5 × 2.5)	1.74(0.18 ~ 8.75)	58 (10 ~ 95)	1.439(3.6 ~ 8355.0)
Melanoma	23	1.2 × 1.0(0.8 × 0.3 ~ 2.5 × 1.6)	1.26(0.24 ~ 4.00)	75 (20 ~ 95)	1.614(103.3 ~ 8281.3)
Pancreas	45	1.5 × 1.0(0.6 × 0.5 ~ 3.5 × 2.2)	1.58(0.30 ~ 7.70)	55 (10 ~ 90)	1.687( 85.5 ~ 6208.4)
Kidney	51	2.7 × 1.0(1.0 × 0.6 ~ 3.5 × 2.3)	2.79(0.60 ~ 8.05)	71 (15 ~ 95)	1.953( 197.3 ~ 7295.2)
Urinary bladder	62	2.1 × 1.0(1.2 × 0.3 ~ 2.8 × 2.3)	2.18(0.36 ~ 6.44)	50 (6 ~ 90)	1.913(60.1 ~ 6922.8)
Others	94	2.1 × 1.0(0.8 × 0.3 ~ 3.5 × 2.5)	2.13(0.24 ~ 8.75)	63 (6 ~ 100)	1.509(9.3 ~ 9809.8)
Total	702	2.0 × 1.0(0.6 × 0.5 ~ 3.5 × 2.5)	2.09(0.18 ~ 8.75)	58 (6 ~ 100)	1.801(3.6 ~ 9809.8)

**Figure 4:**
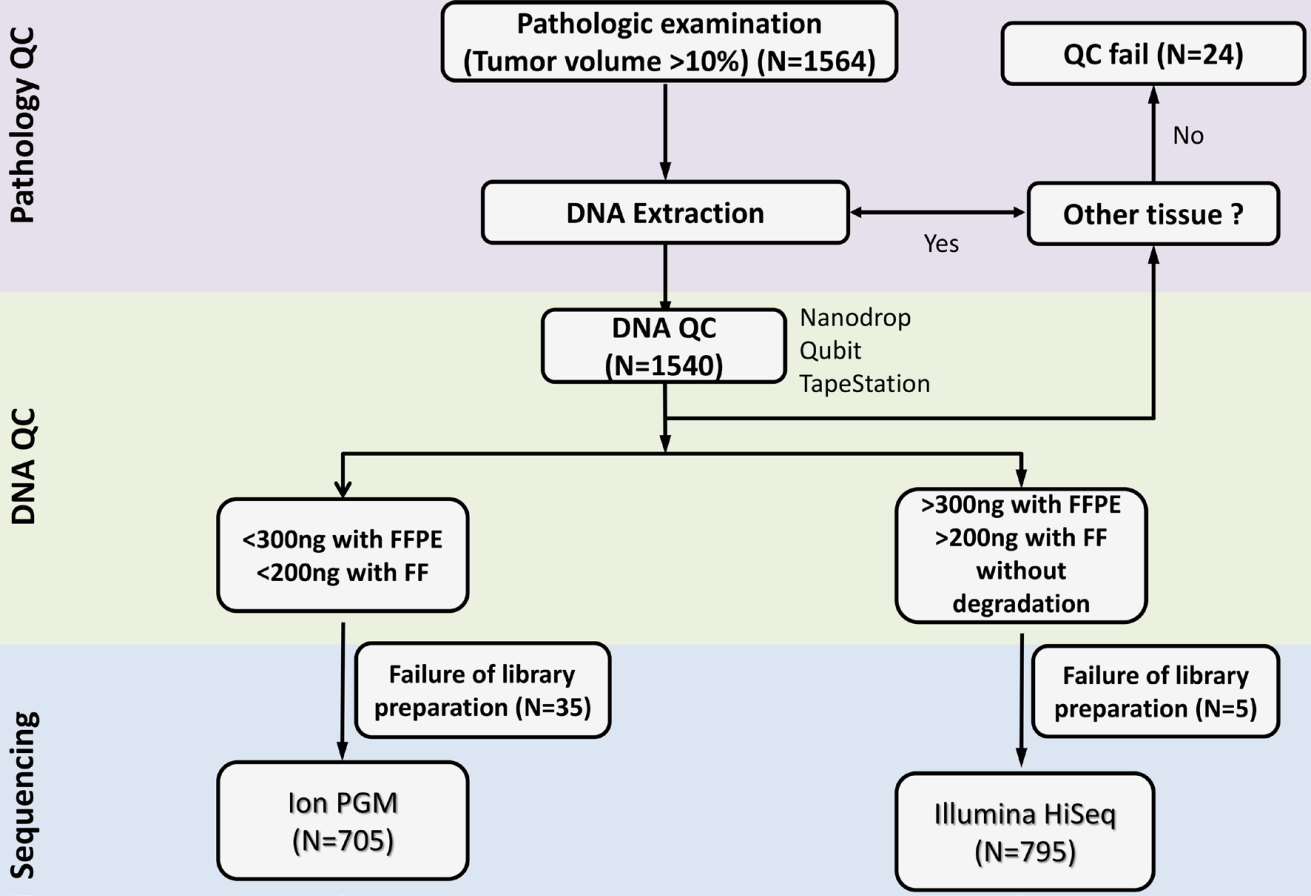
NGS workflow at our institute starting from DNA extraction to quality control and sequencing

## DISCUSSION

Despite the recent technical advances of NGS, tissue sample quality and the fairly large amount of DNA required often limit the sequencing process [11]. Although it is important for pathologists to understand the amount of tissue needed, it is likely even more important for the radiologist or surgeon performing the procedure to acquire the tissue to understand. Indeed, this is a frequent discussion between the clinician and pathologist. To address this issue, we selected successfully sequenced samples and analyzed the submitted sample size and subsequent DNA yield according to the sample acquisition method used as well as the type of cancer.

In experimental models, the NGS illumina platform [10, 12] has been used successfully with as little as 10–70 ng of input DNA, however its use in a clinical setting has been limited. Another platform, the Ion Torrent, has an advantage of low input DNA (10 ng for the Ion PGM cancer hotspot panel) and can run successfully even with low quality DNA [4]. Recently, researchers in a clinical molecular diagnostics laboratory published their experience of a combined workflow using both the MiSeq Illumina and Ion Torrent platforms with the Trueseq amplicon cancer panel and the Ampliseq hotspot panel [12]. They showed a 100% concordance between two panels using a combined workflow. Priority was given to the Tureseq panel for high-quality samples with the Ampliseq panel as a second option. These platforms had a sequencing success rate of 96% when analyzing clinical tumor samples [12]. In our laboratory, we have handled a large number of tissue samples for targeted sequencing using these 2 sequencing platforms for over 4 years with successful genomic results being obtained in over 1000 patients given us a vast amount of data and experience to draw from. In the present study, we particularly focused on the DNA amount needed for successful sequencing.

In our experience, small FF samples > 1 mm^2^ are enough for sequencing, and the mean DNA yield per one endoscopic biopsy piece (8 mm^3^) was 651.7 ng. A previous study that included lung, colon, and skin specimens suggested that 9 mm^3^ of tissue should produce more than 1 μg of DNA in 99% of cases, and our results concur [6]. For FFPE samples, the mean DNA yield per one unstained slide for biopsy and surgical specimens was 114 ng and 1800 ng, respectively. As the minimum DNA requirement varies according to the application or workflow, the tissue requirement cannot be uniformly determined. However, assuming 200 ng as the minimum DNA requirement, a single FF tissue endoscopic biopsy fragment is enough for NGS given the tumor cellularity is > 10%. For NGS test, at least 10% tumor cellularity is usually required due to the background sequencing error rate of the technology and the efficiency of the targeted approach. In the case of FFPE biopsy samples, 3–4 5 μm thick sections should be enough to reach 200 ng.

In the present study, a significant discordance in DNA yield was seen that was dependent on the location of the tumor. FF and FFPE biopsy samples from the lung showed lower DNA yield compared to those from the GI tract. Furthermore, FF samples from lung showed a higher QC failure rate during DNA preparation for NGS. Histologically, lung cancers often show small fragments of viable tissue with large areas of hemorrhage and necrosis, which likely contributes to the lower DNA yield seen in lung specimens. From our present data, EBUS samples from the lung give the lowest yield of DNA. In addition, another likely reason for more frequent pathologic QC failure of lung cancer samples is due to the nature and location of the lung tissue itself. Hemorrhage is a very real complication associated with lung biopsy which can hamper adequate sampling of a lesion. Comparatively, tissue from colorectal cancer is relatively easy to procure given the ease of which the colon can be endoscopically assessed. This will of course lead to larger biopsy sizes and more thoroughly sampled lesions which results in higher DNA yield.

Although fairly extensive with a good sample size, this study has several limitations. First, DNA concentration in the current study was first measured by Nanodrop, and the results of Qubit were not provided in all cases. In general, DNA concentrations measured by Nanodrop are typically higher than Qubit. Qubit is then needed to ensure accurate DNA measurements, especially for degraded DNA from FFPE samples [13]. In many laboratories (including ours), samples are initially checked with Nanodrop and subsequently double checked with Qubit to ensure the accurate measurement of double-stranded DNA [13]. Caution is needed when interpreting DNA concentration of FFPE samples using the Nanodrop assay only. Second, the result of library QC, sequencing QC, and validation results are not provided in the present study and were published previously and In Press.(NEXT, The Oncologist) Input DNA amount can affect library yields and cause amplification bias [5]. In our institute, sequencing results of samples with unsatisfactory QC results were reported with a caveat of the possibility of false positive or negative results. Evaluating and validating variant call results of low quality samples will be explored in a further study.

In summary, we presented our tissue requirements for NGS workflow and shared our experience using a combined NGS workflow. Hopefully, these guidelines and data can be of help to pathologists and clinicians alike in the successful procurement of tissue that will be used in targeted sequencing. In particular, lesions within the lung typically yield lower amounts of DNA, especially EBUS biopsies which may hamper efforts in NGS analysis.

## MATERIALS AND METHODS

### Samples

Between October 2013 and October 2015, a total of 1825 tissue samples were procured from patients with metastatic or recurrent solid cancer to detect genetic alterations for clinical trial enrollment (NCT02299622 - ClinicalTrials.gov. and NEXT-1) [14]. Of the total 1825 samples, 261 freshly obtained operation specimens were excluded from the final analysis due to these types of specimens always providing ample DNA for NGS testing. Of the final 1,564 tissue samples analyzed, 481 (30.8%) were from FF tissue, and 1,083 (69.2%) samples were from FFPE tissue.

### Pathologic quality control (QC)

All FF and FFPE samples (5 μm unstained slides or FFPE blocks) were sent to the Center for Cancer Companion Diagnostics of Samsung Medical Center for pathologic QC. For all FF samples, the number and size of the tissue fragments were measured and recorded. Fresh samples were frozen in liquid nitrogen as quickly as possible after removal from patients and immediately delivered to the laboratory. The tumor tissues were kept in −80°C freezers until DNA extraction.

For both FF and FFPE tissues, 5 μm thick H&E slides were prepared and then analyzed by two experienced pathologists (M.H or S.A) to determine the presence and percentage of tumor cells present. Cases which showed less than 10% tumor were excluded. Tumor-rich areas were marked for manual macro-dissection when tumor percentages were less than 70%, although, macrodissection could not be used in certain cases (small sample size, dispersed tumor cells, or predominance of blood). For FF samples with multiple pieces, pieces without tumor were discarded. For FFPE tissue, 20 unstained slides were prepared, and the pathologists decided on the number of slides to be used for DNA extraction based on size and purity of the tumors. Large areas of necrosis were avoided for analysis. For cases to be included, a minimum tumor percentage of 10% was needed for both FF and FFPE tissues.

### Genomic DNA isolation and quality control

Genomic DNA (gDNA) was extracted using Qiagen DNA FFPE Tissue Kit (Qiagen, Hilden, Germany) or QIAamp DNA Mini Kit (Qiagen, Hilden, Germany) according to the manufacturer's instructions as previously described [15]. RNaseA (Qiagen #19101) was used in all samples. We measured concentration as well as 260/280 and 260/230 nm ratios (ND1000, Nanodrop Technologies, Thermo-Fisher Scientific, MA, USA). Each sample was then further quantified with the Qubit fluorometer (Life Technologies, Carlsbad, California). To estimate DNA degradation, DNA median sizes were measured with a 2200 TapeStation Instrument (Agilent Technologies). The decision of which sequencing panel to use between Ion AmpliSeq^™^and HiSeq CancerSCAN^™^ was made based upon quality and quantity of DNA and any clinical request. The integrated NGS workflow based on DNA amount and QC results is illustrated in Figure 1. If DNA volume is greater than 500 ng by Nanodrop (first step), we further proceed to the Qubit fluorometer. Based on quantity of DNA and the difference with Nanodrop results, we decide on whether to proceed to Illumina HiSeq, Ion PGM, or stop (Figure 1). For Ion PGM, our QC criteria is a DNA concentration less than 1.5 ng/ul as measured with Qubit, or if the difference between Nanodrop and Qubit is more than 10 fold. For HiSeq, the QC criteria is A260/280 > 1.8, A260/230 > 1.8, median size of DNA >15 kb (> 350 bp for FFPE), total DNA amount ~300 ng with FFPE and ~200 ng with FF tissue, and a delta CT < 2.0.

### Cancer panel sequencing

We performed NGS using either Ion AmpliSeq™ Cancer Hotspot Panel v2 with Ion Torrent Personal Genome Machine (PGM, Life Technologies, Grand Island, NY, USA) or a customized cancer panel, CancerSCAN^™^ with Illumina HiSeq 2500 (Illumina, USA) as previously described [16, 17].

Briefly, we constructed libraries using 10 ng of gDNA with the Ion AmpliSeq Library Kit and Ion Xpress Barcodes (Life Technologies) for Ion AmpliSeq^™^. For barcoded library preparations, barcoded adapters from the Ion Xpress Barcode Adapters 1–96 Kit were substituted for the non-barcoded adapter mix in the Ion AmpliSeq Library Kit. Next, the multiplexed barcoded libraries were enriched by clonal amplification using emulsion polymerase chain reaction (PCR) on Ion Sphere Particles (Ion PGMTemplate 200 Kit) and loaded on an Ion 316 Chip. Massively parallel sequencing was carried out on an Ion PGM using the Ion PGM Sequencing 200 Kit v2.

For Illumina HiSeq 2500, we used 250 ng of gDNA after shearing DNA with the Covaris S220 (Covaris, Woburn, MA, USA) and constructed library with customized RNA baits and the SureSelect XT reagent kit, HSQ (Agilent Technologies) as previously described [17]. After enrichment, libraries were multiplexed and sequenced. After the library was hybridized with bait sequences for 16 hours, the captured library was purified and then amplified with an index barcode tag. The quality and quantity of the captured library were measured and sequenced using the 100-bp, paired-end mode of the TruSeq Rapid PE Cluster kit and TruSeq Rapid SBS kit (Illumina, San Diego, CA, USA) as previously described. The results of sequencing and the therapeutic effects of clinical trials have been published [14, 16–18].
